# Change in emotion regulation during the course of treatment predicts binge abstinence in guided self-help dialectical behavior therapy for binge eating disorder

**DOI:** 10.1186/s40337-014-0035-x

**Published:** 2014-12-11

**Authors:** Laurel M Wallace, Philip C Masson, Debra L Safer, Kristin M von Ranson

**Affiliations:** Department of Psychology, University of Calgary, 2500 University Drive NW, Calgary, Alberta Canada; Department of Psychiatry and Behavioral Sciences, Stanford University, Stanford, California USA

**Keywords:** Self-help, Dialectical behavior therapy, Emotion regulation, Binge eating disorder

## Abstract

**Background:**

Dialectical behavior therapy (DBT), which appears to be an effective treatment for binge eating disorder (BED), focuses on teaching emotion regulation skills. However, the role of improved emotion regulation in predicting treatment outcome in BED is uncertain.

**Methods:**

This secondary analysis explored whether change in self-reported emotion regulation (as measured by the Difficulties in Emotion Regulation Scale) during treatment was associated with abstinence from binge eating at post-treatment and 4-, 5-, and 6-month follow-up in individuals who received a guided self-help adaptation of DBT for BED. Participants were 60 community-based men and women with BED who received a self-help manual and six 20-minute support phone calls.

**Results:**

Greater improvement in self-reported emotion regulation between pre- and post-treatment predicted abstinence from binge eating at post-treatment, 4-, 5-, and 6-month follow-up. However, some follow-up results were no longer significant when imputed data was excluded, suggesting that the effect of emotion regulation on binge abstinence may be strongest at 4-month follow-up but decline across a longer duration of follow-up.

**Conclusions:**

This study provides preliminary support for the theoretical role played by improved emotion regulation in achieving binge eating abstinence. If this finding is replicated with larger samples, further research should identify specific techniques to help more individuals to effectively regulate their emotions over a longer duration.

## Background

Binge eating disorder (BED) is associated with specific eating disorder psychopathology, notable psychiatric comorbidity, and considerable psychosocial and functional impairment [1,2]. Furthermore, BED is associated with significant medical morbidity and physical health risks [3,4].

The characteristic feature of BED is persistent binge episodes wherein an unusually large quantity of food is eaten within a discrete period of time, while experiencing a sense of loss of control over eating (i.e., objective binge eating episodes [OBEs]). Thus, an important goal of treatment for BED is to reduce the frequency of such episodes and ultimately promote abstinence from binge eating. Research has demonstrated that numerous psychological treatments may be effective in improving binge eating symptoms, including cognitive behavior therapy (CBT; e.g., [5]), interpersonal psychotherapy (IPT; e.g., [6]), and dialectical behavior therapy (DBT; e.g., [7]). However, although these treatments are effective for many individuals, a significant proportion of individuals still fails to benefit from such treatments [8-10]. For example, randomized controlled trials of group CBT [6], group IPT [6], and group DBT [11] for individuals with BED have indicated that approximately 40% of individuals receiving treatment were not binge abstinent for the past month at one-year follow-up. These findings suggest a need to refine available treatments with the aim of helping more individuals. To do so, further research is needed to clarify the processes involved in treatments with some efficacy and determine predictors of positive treatment outcome.

One psychological mechanism that has been hypothesized to be integral to the development and maintenance of binge eating is maladaptive emotion regulation [12-14]. Emotion regulation is defined as “the extrinsic and intrinsic processes responsible for monitoring, evaluating, and modifying emotional reactions, especially their intensive and temporal features, to accomplish one’s goals” [15]. In essence, emotion regulation involves the ability to tolerate extreme affect and to regulate one’s affect. Binge eating has been described as a maladaptive emotion regulation strategy, wherein binge eating is used to temporarily relieve an aversive emotional state.

Improving emotion regulation is an important goal of DBT as adapted for BED [6]. DBT for BED focuses on understanding and recognizing the link between dysregulated emotions and disordered eating behaviours, and teaching alternative, more adaptive emotion regulation skills. Previous research has demonstrated that DBT is effective in the treatment of BED [7,11]. However, the association of change in emotion regulation with change in binge eating remains uncertain.

### Purpose

In this exploratory study, we conducted secondary analyses of a trial of guided self-help DBT for BED aimed at testing the theory that change in emotion regulation within individuals during the course of treatment is associated with treatment outcome at post-treatment and four-, five-, and six-month follow-up. Treatment outcome was defined as abstinence from binge eating (i.e., zero OBEs for the past 28 days), and was measured at multiple points: post-treatment, four-, five-, and six-month follow-up. By including assessment of outcome at multiple points during follow-up, this study investigated whether any association between change in emotion regulation and subsequent binge eating behaviour remained consistent or changed across time.

### Guided self-help dialectical behavior therapy for binge eating disorder

A guided self-help adaptation of DBT for BED (see [16]) was developed and evaluated in a randomized wait-list controlled treatment trial [17]. In guided self-help treatment, a health worker guides an individual through a self-help manual. Participants in this treatment trial were randomized to receive treatment immediately (immediate treatment condition) or after a three-month waiting period (delayed treatment condition). The current study utilizes data from this treatment trial. Previously reported results from this trial [17] are briefly summarized here so as to provide background context regarding the efficacy of treatment. These results indicated that, at post-treatment, participants assigned to the immediate treatment condition reported significantly improved scores compared to participants assigned to the delayed treatment condition in terms of higher rates of binge abstinence (40.0% versus 3.3%) and fewer past-month OBEs (6.0 versus 14.4), respectively. Participants in the immediate treatment condition additionally demonstrated improved scores on self-report measures of eating psychopathology, anxiety, depression, and quality of life. Furthermore, these participants demonstrated improved emotion regulation at post-treatment, with better emotion regulation scores than those reported by participants in the delayed treatment condition who had not yet received treatment. The association between emotion regulation and treatment outcome (i.e., binge eating) was not examined. Most improvements were maintained at six-month follow-up. Many participants demonstrated a positive response to guided self-help DBT, yet a significant proportion of participants (70.0%) failed to be binge abstinent at follow-up, which is consistent with results from other treatment trials of DBT for BED (e.g., [7,11]).

## Methods

This study received approval from the University of Calgary Conjoint Faculties Research Ethics Board, and informed consent was obtained from all participants prior to participating in the study. A summary of key study variables and procedures is included below; please see Masson and colleagues, 2013 [17] for further details.

### Participants

Sixty participants were recruited from the community via local media, with the study described as a treatment for BED that used a self-help book and telephone support. To participate in the study, individuals were required to meet DSM-IV criteria for BED [18] or subthreshold BED criteria, as assessed via the Structured Clinical Interview for DSM-IV Axis I Disorders – Non-Patient Version [19]. Thus, participants were required to have had an OBE at least once a week, on average, over the preceding six months. Diagnostic criteria in DSM-5 largely aligns with this lower threshold [20]. Exclusion criteria included: involvement in concurrent psychotherapy for binge eating; active psychosis; body mass index (BMI) less than 17.5 kg/m^2^; use of compensatory behaviors at least once per week over the past three months; any change in dose of psychotropic medication over the past three months; and inability to commit adequate time to assessment and treatment (i.e., approximately two to three hours per week for 16 weeks in total). Of 122 potential participants who inquired about the study and were screened, only three (2.5%) indicated that they were unavailable to commit the required amount of time and/or were unavailable for study dates. All participants spoke English fluently and were 18 years or older. Eighty-eight percent (*n*53) of participants were female and 90.0% (*n* = 54) were Caucasian. Age ranged from 24 to 67 years (*M* = 42.8, *SD* = 10.5) and BMI (based on self-reported height and weight data at pre-treatment assessment) ranged from 22 to 69 kg/m^2^ (*M* = 37.9, *SD* = 8.8). All participants had graduated from high school or equivalent. Twenty-five percent (*n* = 15) of participants were taking stable doses of psychotropic medication for mental health issues (primarily depression and/or anxiety symptoms).

Assessment and treatment of both the immediate treatment condition and the delayed treatment condition were conducted identically, with two exceptions. First, participants in the delayed treatment condition began treatment three months after participants in the immediate treatment condition, and therefore completed each assessment (i.e., pre-treatment, post-treatment, and six-month follow-up assessment) three months after participants in the immediate treatment condition had completed the same assessment. Second, participants in the delayed treatment condition completed a baseline assessment at the outset of the study, at the same time that participants in the immediate treatment condition completed a pre-treatment assessment. Thus, participants in the delayed treatment condition were assessed four times (i.e., baseline, pre-treatment, post-treatment, and six-month follow-up), whereas participants in the immediate treatment condition were assessed three times (i.e., pre-treatment, post-treatment, and six-month follow-up). See Table 1. Data from the baseline assessment, which were collected to evaluate treatment efficacy in the randomized wait-list controlled trial, are not considered in the current study.

**Table 1:** **Timing of assessments completed by participants in the immediate treatment condition and delayed treatment condition over 12 months**

**Condition**	**Study timeline**				
	**0 months**	**3 months**	**6 months**	**9 months**	**12 months**
Immediate treatment	#1: Pre-treatment assessment	#2: Post-treatment assessment		#3: 6-month follow-up assessment	
Delayed treatment	#1: Baseline assessment^1^	#2: Pre-treatment assessment	#3: Post-treatment assessment		#4: 6-month follow-up assessment

*Note.* ^1^Baseline assessment data were not included in analyses.

As all participants in both the immediate treatment and delayed treatment condition completed identical treatment, data from participants in both conditions are pooled together for all analyses in the current study. Between-group mediational analyses are not possible with the current dataset due to small sample size and resultant low statistical power, as well as the unbalanced study design (i.e., as the delayed treatment condition began treatment only three months after the immediate treatment condition began treatment, data are not available to compare to the treatment condition’s follow-up data). Preliminary analyses indicated no significant differences between participants in the immediate treatment and delayed treatment condition on OBE frequency or emotion regulation total scores, at pre-treatment, post-treatment, or follow-up; emotion regulation change scores at post-treatment or follow-up; or binge abstinence rates at post-treatment or follow-up (all *p*’s > .1). Lack of group differences in these variables supports our use of pooled data from the immediate treatment condition and the delayed treatment condition in the following analyses.

### Assessment and measures

Each assessment included the Difficulties in Emotion Regulation Scale (DERS; [21]) and items from the Eating Disorder Examination (EDE; [22]). As the EDE assesses eating pathology over the three preceding 28-day intervals, administration of the EDE at six-month follow-up assessment provided eating pathology scores for three time periods (i.e., four, five, and six months after treatment completion). In comparison, administration of the DERS at six-month follow-up assessment provided emotion regulation scores for only one time period (i.e., six months after treatment completion).

### Eating Disorder Examination

The EDE is a well-established semi-structured interview of eating disorder symptomatology. It has established reliability for the assessment of BED [23], and is considered the gold standard for the assessment of eating disorder psychopathology [24]. Only questions relevant to the differential diagnosis of BED were administered (e.g., questions regarding binge eating frequency, behaviors and feelings associated with binge eating, the use of compensatory behaviors, and self-reported height and weight).

The EDE rater was not involved in treatment delivery and was blind to participants’ group assignment at pre-treatment and post-treatment assessment. The rater received training in administration of the EDE prior to the outset of the study and administered the EDE under the supervision of a psychologist. Inter-rater reliability of the EDE binge eating assessment was examined among 12% of the interviews conducted. The original rater’s rating of the frequency of binge eating within the last 28 days and consensus coding of two eating disorder researchers were identical (*r* = 1.0).

### Difficulties in Emotion Regulation Scale

The DERS is a 36-item self-report questionnaire designed to assess multiple aspects of emotion regulation. The Total Score was analysed, which is the sum of six subscale scores: Nonacceptance of Emotional Responses, Difficulties Engaging in Goal Directed Behavior, Impulse Control Difficulties, Lack of Emotional Awareness, Limited Access to Emotion Regulation Strategies, and Lack of Emotional Clarity. Higher scores indicate greater difficulty in emotion regulation (*range*=36-180). Based on pre-treatment data, the DERS demonstrated high internal consistency in the current study (α=.95).

### Treatment

Treatment was administered via a DBT for BED self-help manual developed for this study and six 20-minute support phone calls. In addition, one in-person, 45-minute orientation session was provided at the outset of treatment wherein the self-help manual was distributed and the basic treatment tenets were discussed. The orientation and all support calls were provided by a doctoral candidate in clinical psychology working under the supervision of a psychologist. The manual was designed to be used over thirteen weeks, and the six support calls were provided approximately every two weeks across this treatment period. Each chapter in the manual had a specific focus and included homework that was intended to be worked on by participants during the week that the chapter was read. Support sessions focused on encouraging participants’ use of the manual, answering any questions that participants had about the manual, and problem-solving with participants to determine how they could find the time to use the manual and/or remember strategies discussed in the manual. The manual provided education on three skills modules to aid in regulating emotions (i.e., Mindfulness, Distress Tolerance, and Emotion Regulation), and guided the reader through activities and exercises designed to increase familiarity with these skills and encourage implementation of these skills in daily life.

### Statistical analyses

The ability of post-treatment emotion regulation change scores to predict treatment outcome (i.e., binge abstinence) at post-treatment and four-, five-, and six-month follow-up was examined. An emotion regulation change score at post-treatment was created by calculating the difference between each participant’s post-treatment DERS total score and his/her pre-treatment DERS total score.

Binary logistic regression analyses were used to examine whether DERS post-treatment change scores (i.e., change in emotion regulation ability) predicted binge abstinence at four-, five-, and six-month follow-up. *T*-tests additionally compared the magnitude of DERS post-treatment change scores between participants who were versus were not binge abstinent at post-treatment, four-, five-, and six-month follow-up.

All statistical analyses were compared to a significance level of *p* < .05.

### Missing data

Twenty-one of 60 participants (35.0%) did not fully complete post-treatment assessment (i.e., complete both the EDE and DERS); 27 of 60 participants (45.0%) did not fully complete follow-up assessment. The conservative last observation carried forward method was used in all analyses.

## Results

Mean scores for DERS total and subscale scores are presented in Table 2 for descriptive purposes. Only DERS total scores are included in the following results.

**Table 2:** **Mean emotion regulation scores at pre-treatment, post-treatment, and six-month follow-up (** ***N*** **=60)**

**Difficulties in emotion regulation scale**	**Pre-treatment** ***M*** **(** ***SD*** **)**	**Post-treatment** ***M*** **(** ***SD*** **)**	**6-month follow-up** ***M*** **(** ***SD*** **)**
Total score	102.03 (25.39)	84.04 (25.37)	81.76 (26.35)
Nonacceptance of emotional responses	17.29 (6.57)	13.68 (6.10)	13.25 (6.25)
Difficulties engaging in goal directed behavior	16.15 (4.71)	13.58 (4.48)	13.07 (4.65)
Impulse control difficulties	15.90 (6.26)	12.87 (5.32)	12.50 (5.54)
Lack of emotional awareness	18.48 (4.67)	15.78 (4.88)	15.58 (4.78)
Limited access to emotion regulation strategies	20.51 (8.09)	16.59 (7.50)	16.37 (7.85)
Lack of emotional clarity	13.60 (3.95)	11.73 (3.64)	11.20 (3.64)

Binary logistic regression analyses indicated that DERS change scores at end of treatment predicted binge abstinence at end of treatment (*χ*^*2*^(1) = 6.99, *p* < .01; OR = 1.04, CI = 1.01-1.07). DERS change scores at end of treatment also predicted binge abstinence at each follow-up point: four-month (*χ*^*2*^(1) = 9.23, *p* < .01; OR = 1.06, CI = 1.02-1.10), five-month (*χ*^*2*^(1) = 6.32, *p* < .05; OR = 1.04, CI = 1.01-1.07), and six-month follow-up (*χ*^*2*^(1) = 6.43, *p* < .05; OR = 1.04, CI = 1.01-1.07).^a^ The odds ratios indicated that for every unit increase in DERS post-treatment change score, there was an approximately 4-6% increase in the odds of being binge abstinent at post-treatment and various follow-up points.

In addition, *t*- tests further demonstrated that participants who were binge abstinent at end of treatment reported greater change in emotion regulation at end of treatment, as compared to individuals who were not binge abstinent at end of treatment. Likewise, participants who were binge abstinent at four-, five-, and six-month follow-up also reported greater change in emotion regulation at end of treatment. Specifically, *t-*tests illustrated that participants who were binge abstinent at post-treatment and each follow-up point demonstrated, on average, approximately three times as much change in DERS scores at end of treatment as individuals who were not binge abstinent at those respective time-points (top of Table 3). Effect sizes (Cohen’s *d*) were all approximately large, although the effect size at four-month follow-up (*d* = 1.01) was notably greater than effect sizes at other time points.

**Table 3:** **Mean change in emotion regulation scores among participants who were binge abstinent versus non-abstinent at each follow-up**

	**Abstinent at post-treatment**			**Abstinent at 4-month follow-up**			**Abstinent at 5-month follow-up**			**Abstinent at 6-month follow-up**		
	**Full sample, with imputed data (** ***N*** **=60)**											
	**Yes**	**No**	***t; Cohen’s d***	**Yes**	**No**	***t; Cohen’s d***	**Yes**	**No**	***t; Cohen’s d***	**Yes**	**No**	***t; Cohen’s d***
	**(** ***n*** ** = 23)**	**(** ***n*** ** = 37)**		**(** ***n*** ** = 26)**	**(** ***n*** ** = 34)**		**(** ***n*** ** = 26)**	**(** ***n*** ** = 34)**		**(** ***n*** ** = 24)**	**(** ***n*** ** = 36)**	
DERS post-treatment change	−30.19 (27.20)	−10.39 (19.91)	−3.25*; .83	−31.19 (30.03)	−7.88 (12.97)	−3.70*; 1.01	−28.27 (26.91)	−10.12 (19.96)	−3.00*; .77	−29.12 (28.45)	−10.56 (18.92)	−3.04*; .77
	**Treatment completers only, without imputed data (** ***N*** **=33-39)**											
	**Yes**	**No**	***t; Cohen’s d***	**Yes**	**No**	***t; Cohen’s d***	**Yes**	**No**	***t; Cohen’s d***	**Yes**	**No**	***t; Cohen’s d***
	**(** ***n*** ** = 19-23)**	**(** ***n*** ** = 17-18)**		**(** ***n*** ** = 18-20)**	**(** ***n*** ** = 15-17)**		**(** ***n*** ** = 18-20)**	**(** ***n*** ** = 15-17)**		**(** ***n*** ** = 16-18)**	**(** ***n*** ** = 17-19)**	
DERS post-treatment change	−31.57 (27.01)	−16.79 (24.98)	−1.75; .57	−38.89 (32.44)	−12.26 (13.75)	−3.16*; 1.07	−34.67 (29.22)	−17.33 (25.87)	−1.79; .63	−36.75 (31.23)	−17.41 (23.22)	−2.03; .70

*Note.* DERS = Difficulties in Emotion Regulation Scale. *p < .01.

### Post hoc analyses

In post hoc binary logistic regression analyses, we explored whether attrition and use of imputed data affected results. These analyses included only those participants who had completed both the DERS and the EDE at each time point (i.e., 39 participants at post-treatment and 33 participants at each follow-up point). Overall, results from these “completer” analyses trended in the same direction; however, several results were no longer statistically significant. Specifically, DERS post-treatment change scores predicted binge abstinence at four-month follow-up (*χ*^*2*^(1) = 5.12, *p* < .05; OR = 1.05, CI = 1.01-1.10). Analyses that examined whether DERS post-treatment change scores predicted binge abstinence at post-treatment (*χ*^*2*^(1) = 2.63, *p* = .11; OR = 1.03, CI = 1.00-1.06), five-month follow-up (*χ*^*2*^(1)=2.71, *p* = .10; OR = 1.03, CI = 1.00-1.06), and six-month follow-up (*χ*^*2*^(1) = 3.30, *p* = .07; OR = 1.03, CI = 1.00-1.06) did not reach statistical significance. Likewise, comparison of mean DERS post-treatment change scores between participants who were binge abstinent at post-treatment, four-, five-, and six-month follow-up, and individuals who were not binge abstinent at those respective time points, suggested that participants who were abstinent experienced greater change in emotion regulation from pre-treatment to post-treatment. However, the size of difference in amount of change between participants who were and were not abstinent only reached statistical significance at four-month follow-up (bottom of Table 3). Effect sizes from these analyses were medium at post-treatment, five-, and six-month follow-up, and large at four-month follow-up.

## Discussion

This study explored the predictive significance of magnitude of change in emotion regulation during the course of treatment on binge abstinence at post-treatment, four-, five-, and six-month follow-up among individuals who received guided self-help DBT for BED. In analyses of the full sample, the amount of change in emotion regulation over treatment was associated with binge abstinence at post-treatment, four-, five-, and six-month follow-up. Specifically, the amount of change reported in emotion regulation from pre-treatment to post-treatment was almost three times greater among participants who were binge abstinent as compared to participants who were not binge abstinent. These findings support the theoretical notion that improving emotion regulation is associated with binge abstinence. Such findings, if replicated, may help further improve existing therapeutic efficacy and help more individuals to successfully recover from BED.

It is noteworthy that results shifted when only participants who had completed the full assessment at each time-point were included (i.e., completer analyses, with no imputed data included). In these post hoc completer analyses, the amount of change in emotion regulation from pre-treatment to post-treatment was only predictive of binge abstinence at four-month follow-up; results trended toward but did not attain significance at post-treatment, five-, and six-month follow-up. It is possible that the lack of significant differences in these analyses of the completer sample was attributable to low statistical power (i.e., only 33–39 participants were included in these analyses). With a larger sample, the difference in amount of post-treatment change in emotion regulation between participants who were and were not binge abstinent at each time point may have reached statistical significance whether or not data was imputed. However, these findings could also suggest that an association between change in emotion regulation and treatment outcome appears only during the few months subsequent to treatment completion, and is lost over a longer duration of follow-up. Even in analyses of the full sample, the association between change in emotion regulation and binge abstinence was notably stronger at four-month follow-up than any other assessment point, as indicated by both the odds ratio in regression analyses and Cohen’s *d* effect sizes in *t*-tests. From this perspective, findings indicate the importance of identifying strategies to strengthen the association between change in emotion regulation and treatment outcome for a longer duration, and/or identifying other factors beyond change in emotion regulation that yield sustained positive treatment outcome. As binge eating is a multiply determined behavior that involves numerous interacting variables in its etiology and maintenance, improving emotion regulation may increase the likelihood of binge abstinence but there are likely additional factors that need to be considered in sustaining long-term maintenance of abstinence.

Further research with larger samples is needed. In addition, we encourage research that includes an active comparison group, considers other ways of measuring emotion regulation, and explores additional predictors of treatment outcome.

The current findings need to be interpreted in the context of existing literature. In particular, a randomized controlled trial comparing group DBT to an active comparison group therapy [11] reported significant group differences between OBE frequency and abstinence rates at post-treatment that were not maintained over the 12 month follow-up period. Improvements in in emotion regulation were also similar between treatment groups. In light of these findings, the authors suggested that nonspecific therapeutic factors common to both treatments may account for treatment outcome, rather than specific effects of DBT. When these results are considered alongside results from the current study, it appears that change in emotion regulation *is* important in improving outcome for individuals with BED, although (1) therapeutic approaches other than DBT may also effectively improve individuals’ emotion regulation, (2) DBT may not effectively improve emotion regulation and reduce binge eating for all individuals, and/or (3) the importance of improved emotion regulation for reducing binge eating may vary over time. It is also possible that differences in findings across studies regarding the role of emotion regulation in treatment outcome are attributable to differences in study methodology. For example, analyses in the current study focused upon comparing treatment responders versus non-responders, rather than comparing treatment conditions. Recognizing the substantial portion of individuals who do not respond to BED treatment, analyses that consider differences in individual response within a treatment condition may be particularly important. In addition, in the current study we examined *change* in emotion regulation, rather than focusing on *level* of emotion regulation, to test the hypothesis that magnitude of change across treatment in one’s ability to regulate his/her emotions is predictive of treatment outcome. Furthermore, guided self-help DBT as provided in the current study involved considerably less direct clinical contact over the course of treatment than either group DBT or the active comparison group therapy (i.e., two hours individual phone contact in guided self-help DBT versus 40 hours face-to-face group contact in DBT and the active comparison group), which may have limited the opportunity for nonspecific therapeutic factors (e.g., therapeutic alliance) to contribute to treatment outcome in the current study.

In sum, results from the current study tend to be consistent with the theoretical notion that improved emotion regulation is associated with better treatment outcome in BED. Notably, in the context of a guided self-help treatment, the present findings showed that individuals with BED can learn adaptive emotion regulation skills in a cost-effective way that does not demand significant time with a therapist. However, further research is needed to better understand inconsistencies in results within this study (e.g., between analyses that did and did not use imputed data) and across studies, and to clarify the duration of time after the end of treatment that this association between change in emotion regulation and treatment outcome is strongest. In addition, research is needed to examine the extent to which nonspecific factors associated with general therapeutic and/or natural recovery processes may additionally contribute to treatment outcome. Finally, research should explore whether improved emotion regulation may be a process that influences treatment outcome in other effective psychological treatments for BED, so as to determine the specificity of the effect. Dismantling studies that identify specific techniques used in DBT that contribute most to improved emotion regulation would also be beneficial, as well as research that identifies other treatment orientations beyond DBT that impact emotion regulation.

Strengths of this study include that it used prospective data collected within a randomized controlled trial. Participants were recruited from the community using very few exclusion criteria, which increases the study’s external validity. Measurement of the primary outcome – binge eating – involved a reliably administered, well-established semi-structured diagnostic interview. In addition, measurement of binge eating occurred across multiple points during follow-up. A major limitation of this study is that power to detect significant effects may have been limited by small and unequal cell sizes, particularly in post-hoc analyses that excluded imputed data. Relatedly, attrition and the use of imputed data may have affected results. In addition, a self-report measure was used to assess emotion regulation. In addition, as between-group analyses comparing change in the treatment condition to change in the delayed treatment condition were not possible due to this study’s design, findings indicate that change in emotion regulation may be an important process in positive treatment outcome but do not confirm that change in emotion regulation is specific to DBT. Rather, change in emotion regulation could predict positive treatment outcome among other therapeutic orientations, be part of a natural recovery process, or both. Finally, it is possible that change in emotion regulation, as measured by the DERS, is reflective of change in other factors (e.g., change in general psychopathology). If so, the association between change in emotion regulation and subsequent binge abstinence may be accounted for by change in other factors not examined in this study.

## Conclusions

Overall, this study suggests that change in emotion regulation by the end of treatment predicts binge abstinence in guided self-help DBT for BED. These findings suggest that emotion regulation is an important skill that predicts positive treatment outcome for BED. However, questions remain regarding the strength of the effect of emotion regulation on outcome at various points across time. Replication and further examination of these exploratory findings with larger samples is needed. Further research that considers how to refine and improve upon treatments for BED should explore the extent to which emotion regulation can be emphasized within treatment protocols, consider additional strategies that can be used to enhance emotion regulation across a range of individuals with BED, and consider other mechanisms that may additionally account for positive treatment outcome at post-treatment and over time.

## Endnote

^a^Controversy exists as to whether abstinence from binge eating is the most appropriate criterion of treatment outcome and should be required for remission from BED and other eating disorders [24]. Thus, we also ran a linear regression analysis between DERS post-treatment change scores and OBE frequency scores, thereby assessing treatment outcome among individuals regardless of abstinence status. DERS change scores predicted OBE frequency at post-treatment, four-, five-, and six-month follow-up (*p*’s < .05). Thus analysis of OBE frequency and binge abstinence yielded similar results.

## Abbreviations

BED: Binge eating disorder
CBT: Cognitive behavior therapy
DBT: Dialectical behavior therapy
DERS: Difficulties in Emotion regulation scale
EDE: Eating Disorder Examination
IPT: Interpersonal psychotherapy
OBE: Objective binge eating episode
